# Dislocation of the hip after normal sonographic screening examination: a case report and literature review

**DOI:** 10.1308/rcsann.2023.0107

**Published:** 2024-03-06

**Authors:** A Rehm, R Clegg, P Linardatou Novak, R Shehata

**Affiliations:** Cambridge University Hospitals NHS Foundation Trust, UK

We read with interest the publication by Walton and colleagues.^1^ Contrary to the authors’ claim, their case does not demonstrate that hip subluxation occurred after a normal ultrasound, because (a) the ultrasound images do not show normal hip anatomy according to Graf,^2^ (b) the patient did not have documented dynamic stress testing of the hip during the ultrasound to test if the hip was stable or unstable and (c) the authors did not consider the percentage of femoral head covered by the bony roof/acetabulum (roof percentage/‘50% rule’).^3,4^

**Figure 1:**
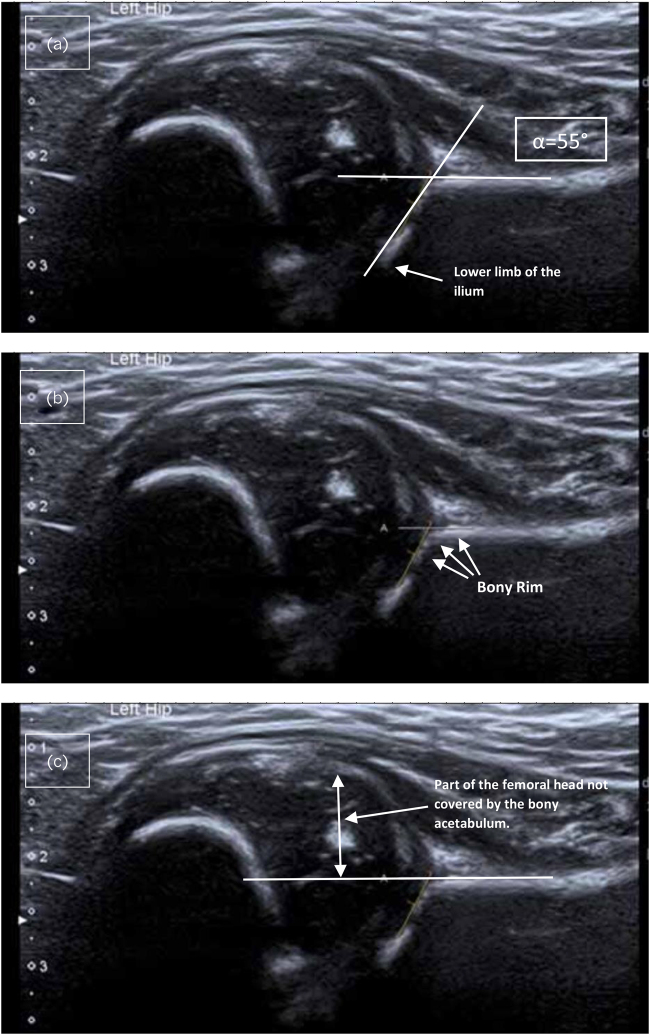
All three hip US images are copies of Walton *et al*’s original Figure 1b, which we use to illustrate why the presented hip should not have been described as normal at 11 weeks of age: (a) we remeasured the alpha angle as 55°, contrary to Walton *et al*’s measured angle of 62°; (b) the bony rim should be angular/sharp or blunt, but here it is instead well rounded, indicating immaturity and (c) more than 50% of the femoral head is not covered by the bony acetabulum (distance indicated by the arrow). The medial extent of the femoral head within the acetabulum is not clearly visible to US, with US having the impression that the femoral head was already subluxed US = ultrasound.

Walton *et al* based their description of a normal hip purely on a static Graf alpha angle,^1^ which they measured as 62°, not considering all criteria defined by Graf,^2^ which have to be met to be able to apply the Graf classification. We measured the alpha angle as 55° (Figure 1a), which we think is more in line with the measurement technique described by Graf.^5^

The bony rim (turning point) is the junction between acetabular concavity and the convexity of the ilium. A type I hip has a well-defined triangular, cuspidal bony rim (it is acceptable for it to be slightly blunt),^2^ with the cartilage roof covering the femoral head. A type II has a rounded bony rim that is ill defined. The images provided by Walton *et al*. show such a rounded junction,^1^ with a large amount of the femoral head not being covered by the cartilage roof, therefore clearly showing that the hip was underdeveloped at 11 weeks (Figure 1b).

Morin *et al* reported that a femoral head coverage (FHC) by the bony acetabulum of >58% was associated with no clinical abnormalities and consistent normal acetabular indices.^6^ Terjesen *et al* identified a FHC of 50% to be the threshold between normal and potentially abnormal hips.^3^

Harcke and Pruszczynski reiterated the rational of the 50% rule of FHC to assess acetabular development.^4^ Walton *et al*’s ultrasound images show a FHC of not more than 40% (Figure 1c),^1^ supporting that the hip was abnormal and unstable at the time, which is supported by the fact that the hip was already dislocated at the five months clinic presentation.

Alshameeri and Rehm reported a case similar to the latter,^7^ where ultrasound images showed instability with subluxation of the femoral head, which were reported as normal, probably because the alpha angle measured >60°.

We conclude that the hip ultrasound images presented by Walton *et al* do not show a normal hip,^1^ which highlights the importance of adhering to the ultrasound standards (measurement of angles and anatomic landmarks) as described by Graf, to examine for stability and to consider the 50% rule of FHC.^3,4,6^ These standards help to identify underdeveloped and dysplastic hips early, which can be treated easily with a Pavlik harness, possibly avoiding dislocation and the associated more invasive and, for children and care givers, more involving higher risk treatments.^1,7^
